# Comment on Gatti et al. Simultaneous Bilateral Reconstruction of Chronic Achilles Tendon Rupture with Flexor Digitorum Longus Transfer and Turndown Flaps: A Case Report and Review of Literature. *J. Clin. Med.* 2026, *15*, 922

**DOI:** 10.3390/jcm15072666

**Published:** 2026-04-01

**Authors:** Filippo Spiezia, Francesco Oliva, Nicola Maffulli

**Affiliations:** 1 Department of Health Sciences, University of Basilicata, 85100 Potenza, Italy; 2 Department of Orthopaedics and Trauma Surgery, San Carlo Hospital, 85100 Potenza, Italy; 3 Department of Sport Traumatology, Università Telematica San Raffaele, 00166 Rome, Italy; 4 Department of Trauma and Orthopaedic Surgery, University La Sapienza, 00185 Rome, Italy; 5 Centre for Sports and Exercise Medicine, Barts and the London School of Medicine and Dentistry, Mile End Hospital, Queen Mary University of London, London E1 4DG, UK

We read with great interest the work by Gatti SD et al. [1], and we congratulate the Authors for the interesting case report and review of the literature.

It is true that transferring the tendon of peroneus brevis can result in lower eversion strength, as we have indeed reported [2]. A major concern regarding transfer of the tendon of the peroneus brevis is the reduced strength of plantar flexion and the eversion of the ankle [3]. The peroneal muscles provide only 4% of the total work capacity in plantar flexion, and the peroneus brevis provides 28% of the eversion capacity of the hindfoot [4].

However, the side-to-side difference in eversion strength, although statistically significant, is of dubious clinical relevance: we have not observed a higher rate of, for example, inversion injury of the ankle in patients in whom this tendon was used as a graft for chronic Achilles tendon ruptures [2], and, to our knowledge, this has not been ever reported.

Gatti et al. also stated the following:

“Specifically, in a single-stage bilateral reconstruction, utilizing hamstring autografts would have required a change in the patient’s intraoperative positioning, increasing the technical complexity and prolonging the cumulative operative time” [1].

We reported the use of gracilis for chronic tears of the Achilles tendon [5], in which it was clearly stated that the harvest took place with the patient prone through a vertical 2.0–2.5 cm longitudinal incision over the pes anserinus. A similar technique was described when harvesting the semitendinosus tendon [6]; again, the tendon of the semitendinosus is harvested through a vertical 2.5–3 cm longitudinal incision over the pes anserinus, with the patient prone. We never advocated that the hamstring tendons are harvested with the patient supine, and that, after the harvest, the patient is positioned prone. We also point out that, following the original articles where we described the harvest of the hamstring tendons from the pes anserinus with the patient prone [5,6], we have described, and now routinely perform, harvesting such tendons through a small transverse incision in the popliteal fossa [7].

The decision to utilise a tendon transfer rather than a passive free graft was based on the functional status of the gastrocnemius–soleus complex [8]. In long-standing ruptures, irreversible muscle atrophy and fatty infiltration often compromise the contractile potential of the original muscle after 3 to 6 months [9].

While a free graft acts merely as a bridge, a local tendon transfer introduces a fresh, functional “motor unit” to actively drive plantarflexion. This is particularly advantageous when the viability of the gastrocnemius is “uncertain”.
